# Association between serum albumin creatinine ratio and all-cause mortality in intensive care unit patients with heart failure

**DOI:** 10.3389/fcvm.2024.1406294

**Published:** 2024-07-04

**Authors:** Jiuyi Wang, Ni Li, Yunkai Mu, Kai Wang, Guibo Feng

**Affiliations:** ^1^ Department of General Medicine, Yongchuan Hospital of Chongqing Medical University, Chongqing, China; ^2^ Department of Cardiology, Chongqing Bishan Hospital of Traditional Chinese Medicine, Chongqing, China; ^3^ Department of Cardiology, The Second Affiliated Hospital of Chongqing Medical University, Chongqing, China

**Keywords:** serum albumin creatinine ratio, intensive care unit, heart failure, mortality, risk stratification

## Abstract

**Background:**

The serum albumin creatinine ratio (sACR) has been established as a potential indicator for heart disease, however, its relationship with prognosis in intensive care unit (ICU) patients with heart failure remains uncertain. This study aimed to investigate the association between sACR levels and all-cause mortality ICU patients with heart failure.

**Methods:**

Clinical data from MIMIC-Ⅳ database was utilized for the analysis of ICU patients with heart failure. Patients were categorized into quartiles (Q1-Q4) based on sACR levels. Kaplan-Meier survival analysis and multivariate adjusted Cox regression models were employed to assess the association between sACR levels and mortality outcomes within 365 days. Subgroup analysis was used to evaluate the prognostic impact of sACR across diverse populations. Restricted cubic spline curves and threshold effect analysis were utilized to quantify the dose-response relationship between sACR levels and risk of all-cause mortality. Mediating effects analysis was conducted to present the involvement of albumin and creatinine in the association between sACR and outcomes.

**Results:**

The analysis encompassed a cohort of 4,506 patients, with Kaplan-Meier curves indicating that individuals with lower sACR levels exhibited an elevated risk of all-cause mortality (log-rank *p* < 0.001). Multivariate adjusted Cox regression and subgroup analysis demonstrated that individuals in Q2 [hazard ratio (HR) 0.82, 95%CI 0.71∼0.96], Q3 (HR 0.76, 95%CI 0.64∼0.91) and Q4 (HR 0.62, 95%CI 0.50∼0.76) had a decreased risk of mortality compared to individuals in Q1 (lower levels of sACR) (*p* for trend < 0.001), and this inverse relationship was consistently observed across various subgroups. Subsequent restricted cubic spline analysis revealed a negative yet nonlinear relationship between sACR and all-cause mortality (*p* for nonlinear < 0.001), and threshold effect analysis indicated an effect threshold of 3.75. Additionally, mediating effects analysis emphasized that sACR influenced the outcome not only through serum albumin and creatinine pathways, but also through direct mechanisms.

**Conclusion:**

The study found that low levels of sACR were independently associated with an increased risk of one-year all-cause mortality in ICU patients with heart failure, with a threshold effect, which could potentially serve as an early warning indicator for high-risk populations.

## 1 Introduction

Heart failure is a condition of considerable importance, distinguished by a high prevalence and mortality rate. In Europe, the one-year mortality rate for hospitalized heart failure patients can be as high as 17% (1), while in the United States, 10%–15% of hospitalized heart failure patients exhibit a more critical state, necessitating intensive care unit (ICU) admission for advanced care (2). Timely and precise risk assessment plays a crucial role in tailoring treatment and management strategies, ultimately enhancing these circumstances (3).

The onset and progression of heart failure are characterized by the activation of neurohormones, inflammatory responses, metabolic dysregulation, and other factors, leading to abnormal levels of biomarkers such as serum albumin and creatinine, especially during acute exacerbations (1). Serum albumin, predominantly produced by hepatocytes, serves crucial functions in the regulation of colloid osmotic pressure, immune responses, and antioxidant mechanisms (4). Hypoalbuminemia has been associated with increased mortality in heart failure patients, possibly due to its reflection of systemic inflammation, malnutrition, and cachexia, which are common in advanced heart failure (5). Serum creatinine, a product of renal metabolism, serves as a significant marker for renal function impairment, Renal dysfunction is a frequent comorbidity in heart failure patients and is strongly associated with adverse outcomes in individuals with acute heart failure (6). Furthermore, renal function influences serum albumin levels, and the serum albumin to serum creatinine ratio (sACR) has been identified as a novel biomarker (7). Given the complex interplay between heart failure, systemic inflammation, and renal dysfunction, integrating serum albumin and creatinine into sACR may offer a more comprehensive assessment of the pathophysiological alterations in heart failure, thus enhancing prognostic accuracy. Prior studies have demonstrated the efficacy of sACR as a prognostic assessment tool in populations including acute myocardial infarction and heart transplant patients (8, 9). However, there remains a dearth of research examining the association between sACR and prognosis in ICU heart failure patients.

Therefore, we hypothesized that sACR may serve as a valuable tool for identifying adverse prognostic outcomes in this population. Our study sought to investigate the association between sACR levels and prognosis in ICU heart failure patients utilizing data from the MIMIC-IV database.

## 2 Methods

### 2.1 Study population

**Figure 1:**
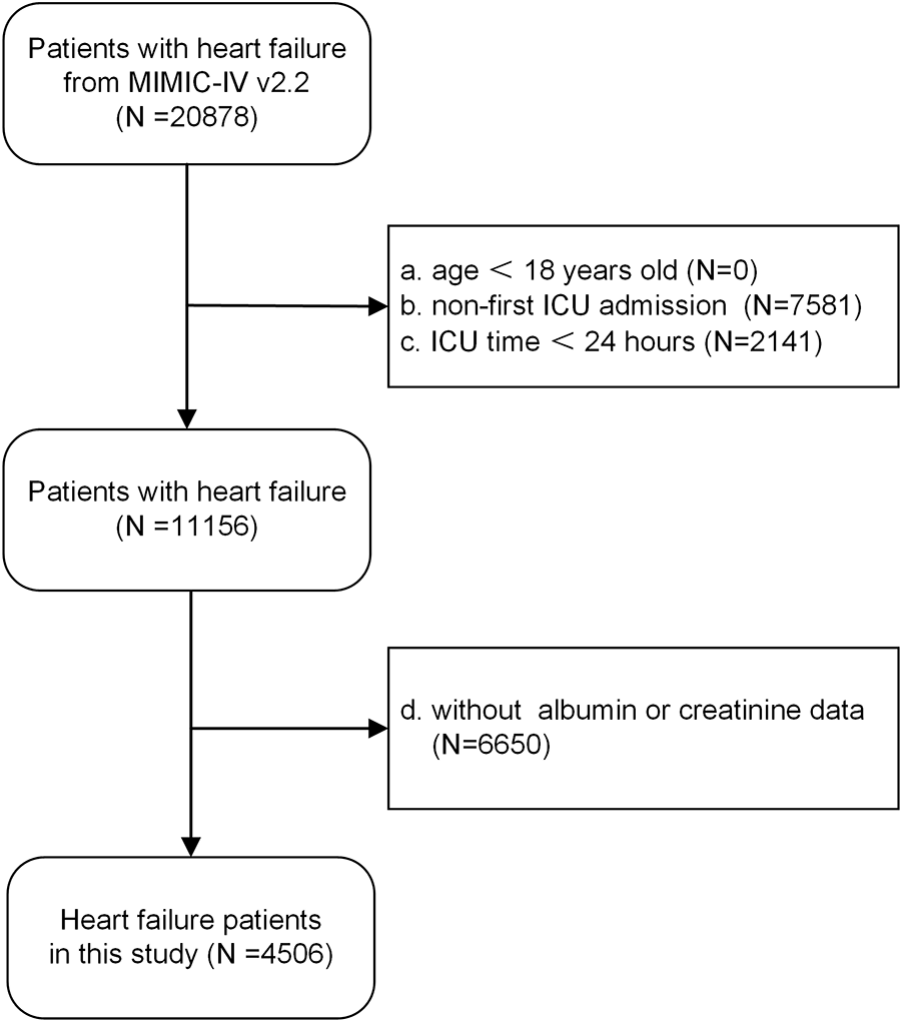
Flow diagram. It presented the inclusion and exclusion of the study participants.

The study population was derived from the Medical Information Mart for Intensive Care IV (MIMIC-IV, version 2.2) database. MIMIC-IV is a comprehensive repository of data from a single medical center encompassing information on more than 190,000 ICU patients treated between 2008 and 2019. Inclusion criteria for this study encompassed all patients diagnosed with congestive heart failure, identified through the use of International Classification of Diseases, Ninth and Tenth Revision (ICD-9 and ICD-10) codes (Supplementary Table S1). Exclusion criteria were applied to exclude those patients: (1) under 18 years of age, (2) not admitted to the ICU for the first time, (3) with an ICU stay of less than 24 h, (4) without serum albumin or creatinine data. Ultimately, as shown in the flowchart (Figure 1), a total of 4,506 patients were recruited for the study. The procedures involving human participants in this study adhered to the ethical standards set forth by the institutional and/or national research council, as well as the 1964 Helsinki declaration and its subsequent amendments or equivalent ethical guidelines. As the study involved secondary analysis of de-identified patient data from a specified dataset, the necessity for obtaining informed consent from participants was waived. Furthermore, due to the public availability of the data and the inability to ascertain the identities of individuals directly or indirectly, ethical review was deemed unnecessary.

### 2.2 Data collection and follow-up

Clinical data were initially gathered within 24 h following ICU admission, encompassing demographic characteristics (gender, weight, height, age), comorbidities [hypertension, diabetes, chronic obstructive pulmonary disease (COPD)], vital signs upon admission [heart rate, respiratory rate, saturation of peripheral oxygen (SpO2) and systolic blood pressure (SBP)], laboratory tests results [hemoglobin, mean corpuscular volume (MCV), mean corpuscular hemoglobin concentration (MCHC), mean corpuscular hemoglobin (MCH), red cell distribution width (RDW), platelets, white blood cell count (WBC), monocytes, neutrophils, total bilirubin, serum albumin, aspartate aminotransferase (AST), alanine aminotransferase (ALT), alkaline phosphatase (ALP), total bilirubin, blood urea nitrogen (BUN), serum creatinine, serum glucose], medication details [dopamine, dobutamine, milrinone, epinephrine, norepinephrine, adrenaline, vasopressin, furosemide, angiotensin-converting enzyme inhibitor/angiotensin Ⅱ receptor blockers (ACEI/ARB), *β*-blockers], mechanical ventilation usage, continuous renal replacement therapy (CRRT), and follow-up data (database provided the mortality within one year for all patients). sACR was calculated using the following formula: sACR = serum albumin (g/dl)/serum creatinine (mg/dl). The utilization of inotropes or vasopressors was defined as the necessary for dopamine, dobutamine, milrinone, phenylephrine, norepinephrine, epinephrine, or vasopressin. The primary endpoint was all-cause mortality within 1 year after discharge.

### 2.3 Statistical analysis

To delineate baseline characteristics between populations with different sACR levels, clinical characteristics and overall mortality rates, stratified by sACR quartiles (Q1: 0.15∼1.38, Q2: 1.38∼2.38, Q3: 2.38∼3.50, Q4: 3.5∼13.00), were compared using appropriate statistical tests such as the Kruskal-Wallis H test, chi-square test, or ANOVA. Normally distributed variables were represented as mean ± standard deviation, while non-normally distributed variables were represented as median (interquartile range), and categorical variables as percentages.

Additionally, to assess the differences in outcome events between populations at different sACR levels, Kaplan-Meier survival analysis and Log-Rank test were utilized to evaluate the risk of the primary endpoint in patients with varying sACR levels.

Then, to minimize confounding factors on sACR and outcomes, sACR was assessed as a categorical (quartiles), ordinal (increase per quartile), and continuous variable (per unit increase) in four distinct Cox regression models to determine the hazard ratio (HR) and 95% confidence interval (CI) for all-cause mortality, as well as conducting trend tests. Model 0: solely incorporated sACR without any covariate adjustment; Model 1: adjusted for demographic characteristics (age, gender) and comorbidities (diabetes, hypertension and COPD); Model 2: adjusted for the variables in model 1 plus situation of inflammation and anemia (neutrophils, hemoglobin), dysfunction of liver and kidney (albumin, total bilirubin, BUN, creatinine); Model 3: adjusted for the variables in model 2 plus use of medications and instrument (ACEI/ARB, *β*-blockers, inotropes or vasopressors, CRRT, mechanical ventilation). The lowest quartile of sACR served as the reference category for all models.

Subsequently, to evaluate the prognostic significance of sACR in various populations, subgroup analyses and interaction tests were conducted utilizing Model 3. The analyses took into account various demographic and clinical factors, including age, gender, presence of hypertension, diabetes, and other comorbidities, as well as utilization of inotropes and vasopressors, CRRT, and mechanical ventilation.

Next, to capture the dose-response relationship between sACR and all-cause mortality, restricted cubic spline analysis was employed with threshold effect analysis conducted as needed.

Additionally, to investigate whether the effect of sACR on outcomes was achieved through serum creatinine and albumin, mediation analysis was performed to explore the potential mediating effects of albumin and creatinine on the association between sACR and the prognosis of ICU heart failure patients.

Finally, to test the reliability of our main findings, we conducted a sensitivity analysis. Relative risk values for sACR quartiles were obtained using univariate and multivariate modified Poisson regression with all-cause mortality within 1 year as the outcome. All statistical analyses were carried out using R (version 4.3.1), with a significance level of *p* < 0.05.

## 3 Results

### 3.1 Population baseline characteristics

Table 1 presented the baseline characteristics for all 4,506 participants from MIMIC-Ⅳ, categorized by quartiles of sACR. Patients with lower sACR levels demonstrated higher prevalence of diabetes and hypertension, hepatic and renal impairment, CRRT rates, and lower hemoglobin levels (*p* < 0.001 for all). The incidence of endpoint events in the overall population was 43.1%. Patients with lower ACR levels exhibited higher all-cause mortality (*p* < 0.001).

**Table 1:** Baseline characteristics of MIMIC-Ⅳ participants according to quartiles of serum albumin to serum creatinine ratio.

Characteristics	Overall (*n* = 4,506) (0.15∼13.00)	Q1 (*n* = 1,126) (0.15∼1.38)	Q2 (*n* = 1,127) (1.38∼2.38)	Q3 (*n* = 1,126) (2.38∼3.50)	Q4 (*n* = 1,127) (3.50∼13.00)	*p*-value
Age (years), mean (SD)	72.08 (14.38)	70.73 (14.20)	74.18 (13.39)	73.00 (14.39)	70.41 (15.17)	<0.001
Male, *n* (%)	2,537 (56.3)	734 (65.2)	684 (60.7)	643 (57.1)	476 (42.2)	<0.001
BMI (kg/m2), mean (SD)	28.81 (6.71)	29.19 (6.77)	29.15 (6.67)	28.61 (6.61)	28.29 (6.76)	0.002
Diabetes, *n* (%)	1,836 (40.7)	622 (55.2)	500 (44.4)	410 (36.4)	304 (27.0)	<0.001
Hypertension, *n* (%)	3,412 (75.7)	931 (82.7)	877 (77.8)	826 (73.4)	778 (69.0)	<0.001
COPD, *n* (%)	913 (20.3)	198 (17.6)	236 (20.9)	227 (20.2)	252 (22.4)	0.038
Heart rate (/min), mean (SD)	90.53 (21.41)	87.87 (21.40)	90.86 (22.21)	91.03 (20.70)	92.38 (21.08)	<0.001
Resp rate (/min), mean (SD)	20.94 (6.33)	20.64 (5.83)	21.32 (6.84)	20.85 (6.16)	20.93 (6.44)	0.079
SpO2(%), mean (SD)	96.22 (4.59)	96.32 (4.55)	95.89 (5.57)	96.23 (4.35)	96.43 (3.70)	0.033
SBP(mmHg), mean (SD)	121.56 (25.77)	118.56 (27.25)	120.16 (25.47)	121.44 (24.70)	126.06 (25.01)	<0.001
Hemoglobin (g/dl), mean (SD)	10.88 (2.48)	9.90 (2.27)	10.69 (2.49)	11.33 (2.47)	11.58 (2.35)	<0.001
MCH (pg), mean (SD)	29.76 (2.92)	29.79 (2.97)	29.52 (3.01)	29.77 (2.90)	29.95 (2.79)	0.007
MCV (fl), mean (SD)	92.25 (7.83)	93.36 (8.33)	91.88 (7.97)	91.99 (7.50)	91.77 (7.37)	<0.001
MCHC (g/dl), mean (SD)	32.28 (1.78)	31.93 (1.75)	32.15 (1.75)	32.37 (1.76)	32.66 (1.77)	<0.001
RDW (%), mean (SD)	15.75 (2.56)	16.47 (2.67)	15.99 (2.56)	15.54 (2.45)	15.02 (2.33)	<0.001
Platelets (10^9/L), mean (SD)	222.24 (114.43)	214.03 (118.43)	215.40 (113.19)	224.81 (110.06)	234.70 (114.83)	<0.001
WBC(10^9/L), mean (SD)	12.98 (10.22)	13.11 (8.60)	13.43 (10.27)	13.50 (13.61)	11.89 (7.19)	<0.001
Monocytes (10^9/L), mean (SD)	0.72 (0.72)	0.70 (0.69)	0.74 (0.84)	0.75 (0.81)	0.69 (0.49)	0.128
Neutrophils (10^9/L), mean (SD)	10.37 (6.75)	10.93 (7.24)	10.95 (7.29)	10.31 (6.79)	9.30 (5.37)	<0.001
Total protein (g/dl), mean (SD)	5.58 (1.04)	5.32 (1.10)	5.56 (1.00)	5.68 (1.03)	5.77 (0.99)	<0.001
Albumin (g/dl), mean (SD)	3.26 (0.62)	3.03 (0.65)	3.18 (0.59)	3.34 (0.58)	3.51 (0.56)	<0.001
ALT(U/L), mean (SD)	107.04 (430.51)	170.51 (684.21)	122.44 (434.64)	74.77 (201.90)	60.49 (191.50)	<0.001
AST(U/L), mean (SD)	162.19 (714.34)	288.94 (1,248.22)	167.36 (584.77)	105.16 (261.87)	87.37 (222.49)	<0.001
ALP(U/L), mean (SD)	118.65 (113.61)	140.87 (143.92)	118.14 (95.14)	108.05 (93.44)	107.54 (111.53)	<0.001
Total bilirubin (mg/dl), mean (SD)	1.23 (2.39)	1.43 (3.30)	1.26 (1.86)	1.20 (2.41)	1.03 (1.60)	0.001
BUN(mg/dl), mean (SD)	38.15 (27.75)	66.70 (33.59)	40.48 (19.45)	27.13 (12.90)	18.32 (8.54)	<0.001
Creatinine (mg/dl), mean (SD)	1.97 (1.89)	4.25 (2.59)	1.70 (0.39)	1.16 (0.23)	0.77 (0.19)	<0.001
Glucose (mg/dl), mean (SD)	164.58 (103.49)	168.66 (125.45)	177.14 (112.47)	162.80 (87.69)	149.71 (79.96)	<0.001
CRRT, *n* (%)	342 (7.6)	295 (26.2)	36 (3.2)	9 (0.8)	2 (0.2)	<0.001
Ventilation, *n* (%)	1,801 (40.0)	474 (42.1)	457 (40.6)	448 (39.8)	422 (37.4)	0.152
Mortality, *n* (%)	1,944 (43.1)	631 (56.0)	526 (46.7)	451 (40.1)	336 (29.8)	<0.001

BMI, body mass index; COPD, chronic obstructive pulmonary disease; SpO^2^, saturation of peripheral oxygen; SBP, systolic blood pressure; MCH, mean corpuscular hemoglobin; MCV, mean corpuscular volume; MCHC, mean corpuscular hemoglobin concentration; RDW, red cell distribution width; WBC, white blood cell count; ALT, alanine aminotransferase; AST, aspartate aminotransferase; ALP, alkaline phosphatase; BUN, blood urea nitrogen; CRRT, continuous renal replacement therapy.

### 3.2 Kaplan-Meier survival curve

**Figure 2:**
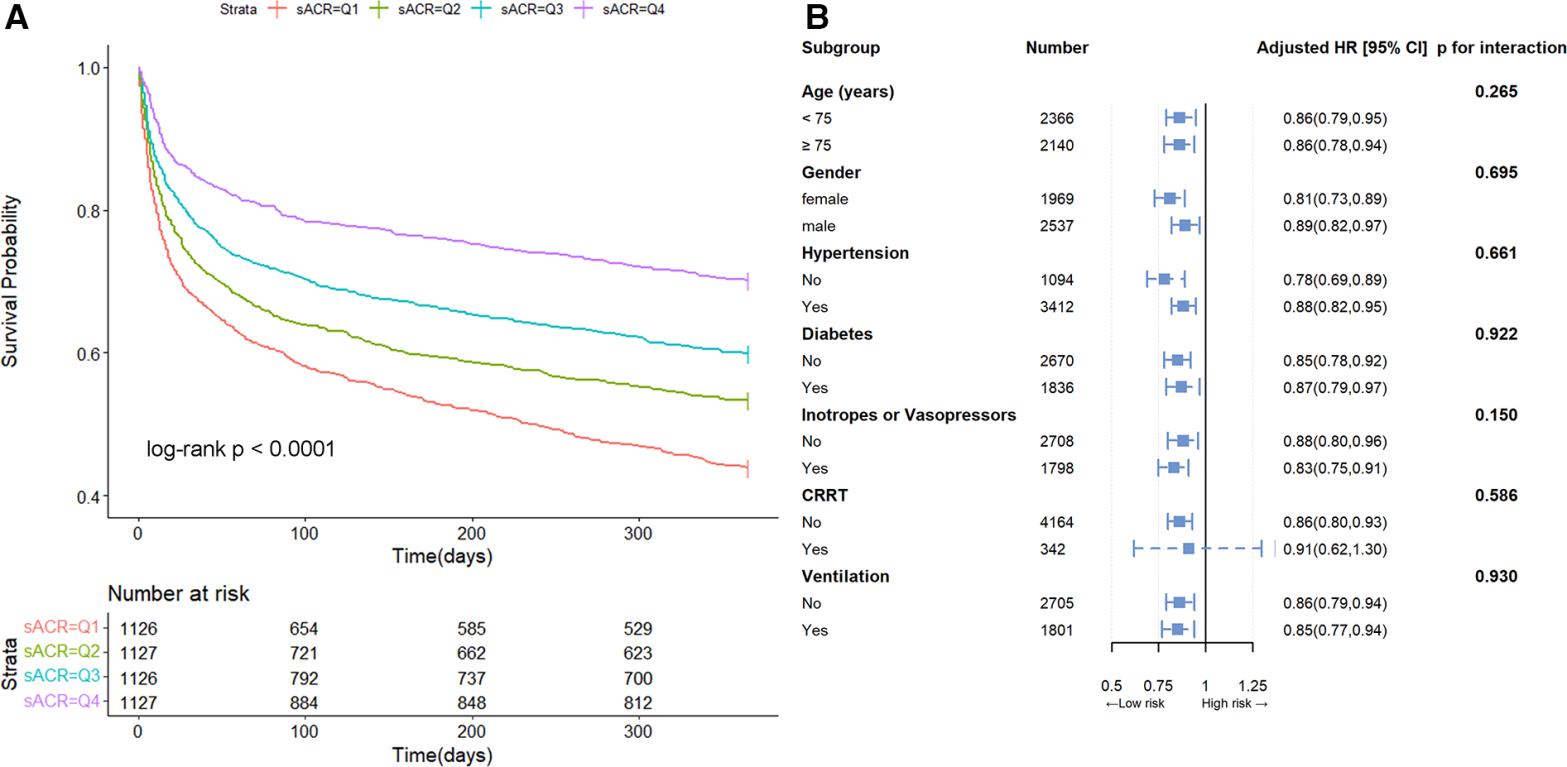
Kaplan-Meier survival curve and subgroup analysis. Cumulative incidence of all-cause mortality according to sACR quartiles (**A**) and hazard ratios and error bars delineating 95% confidence intervals from Model 3 by subgroups (**B**).

Kaplan-Meier survival curve analysis was used to assess the cumulative survival rate. Figure 2A illustrated the one-year cumulative survival rates for the four groups (Q1, Q2, Q3, and Q4) stratified based on sACR quartiles. Patients with lower sACR levels exhibited a significantly deterioratived prognosis compared to those with higher sACR levels.

### 3.3 Adjusted hazard ratios and subgroup analysis

In Model 3, after adjustment for age, gender, diabetes, hypertension, COPD, neutrophils, hemoglobin, albumin, total bilirubin, BUN, creatinine, ACEI/ARB, *β*-blockers, inotropes or vasopressors, CRRT and ventilation, the multivariate HRs for overall mortality in Q2, Q3, and Q4 groups compared to Q1 (reference) were 0.82 (95% CI, 0.71∼0.96), 0.76 (95% CI, 0.64∼0.91), and 0.62 (95% CI, 0.50∼0.76), respectively (*p* for trend <0.001) (Table 2). When sACR quartile was considered as an ordinal variable, each quartile increase in sACR was linked to a 14% reduction in the hazard of mortality following adjustment for multiple variables (HR 0.86, 95% CI, 0.81∼0.92). Furthermore, subgroup analysis demonstrated that this inverse association remained consistent across various subgroups (*p* for interaction > 0.05) (Figure 2B).

**Table 2:** Cox models for the association between serum albumin creatinine ratio and all-cause mortality in one year.

sACR	Case/Total	Model 0	Model 1	Model 2	Model 3
Quartiles		Hazard Ratio(95% Confidence Interval)			
Q1	1,126/4,506	reference	reference	reference	reference
Q2	1,127/4,506	0.77 (0.69,0.87)	0.68 (0.60,0.76)	0.79 (0.68,0.92)	0.82 (0.71,0.96)
Q3	1,126/4,506	0.62 (0.55,0.70)	0.55 (0.49,0.63)	0.72 (0.61,0.86)	0.76 (0.64,0.91)
Q4	1,127/4,506	0.43 (0.38,0.49)	0.39 (0.34,0.45)	0.58 (0.47,0.70)	0.62 (0.50,0.76)
*p* for trend		<0.001	<0.001	<0.001	<0.001
Per quartiles increase	4,506/4,506	0.76 (0.73,0.79)	0.74 (0.71,0.77)	0.85 (0.79,0.90)	0.86 (0.81,0.92)
Per unit increase	4,506/4,506	0.80 (0.77,0.83)	0.81 (0.78,0.84)	0.91 (0.86,0.95)	0.92(0.87,0.96)

Model 0: serum albumin creatinine ratio without adjust; Model 1: age, gender, diabetes, hypertension and chronic obstructive pulmonary disease were adjusted; Model 2: the variables in model 1 plus neutrophils, hemoglobin, albumin, total bilirubin, blood urea nitrogen, creatinine were adjusted; Model 3: the variables in model 2 plus angiotensin-converting enzyme inhibitor/angiotensin Ⅱ receptor blockers, *β*-blockers, inotropes or vasopressors, continuous renal replacement therapy and ventilation were adjusted.

### 3.4 Restricted cubic spline and threshold effect analysis

**Figure 3:**
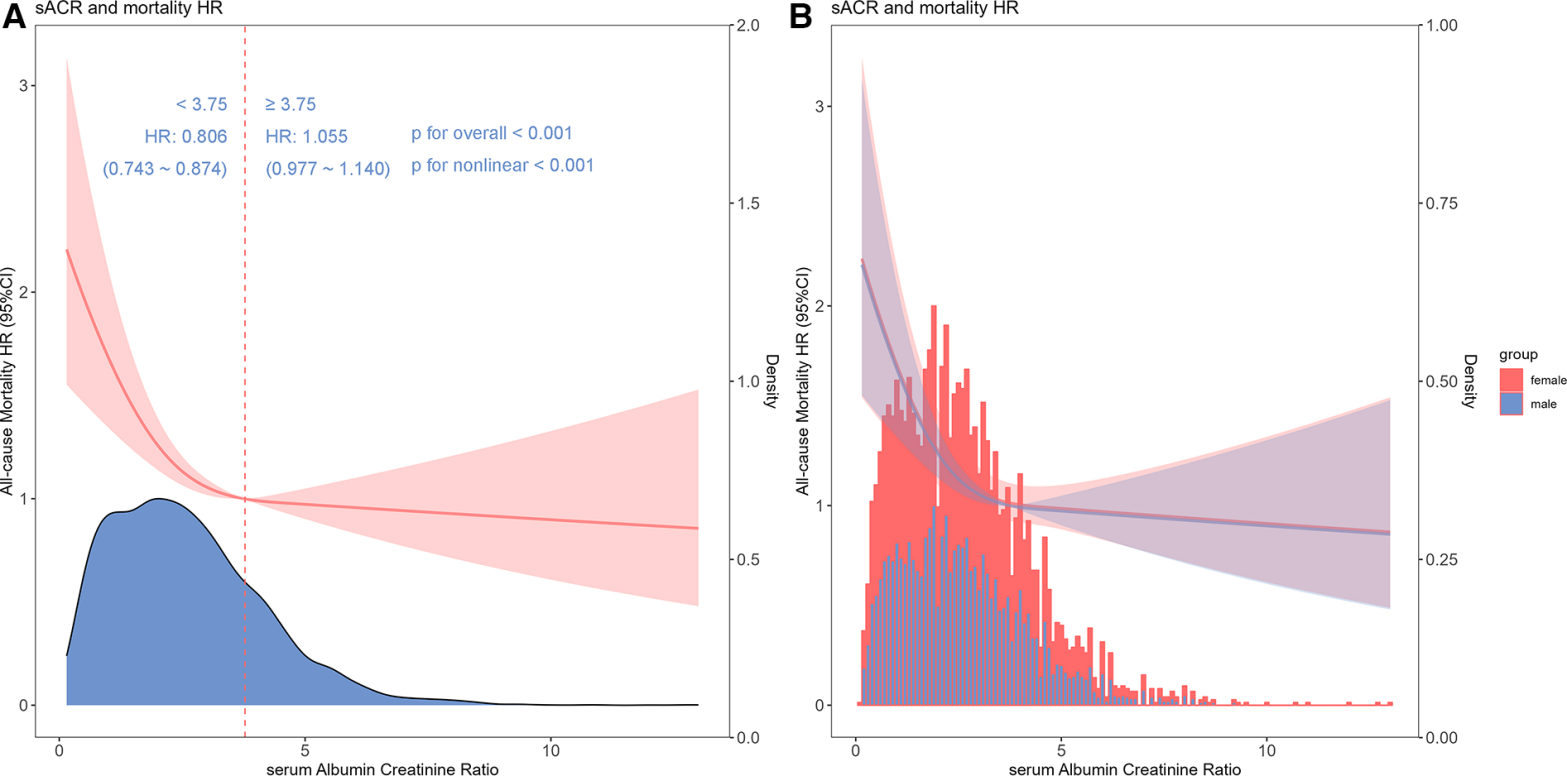
Restricted cubic spline and threshold effect analysis. Cubic spline model of the association between sACR and risk of all-cause mortality in all patients adjusted with age, gender, diabetes, hypertension, chronic obstructive pulmonary disease, neutrophils, hemoglobin, albumin, total bilirubin, blood urea nitrogen, creatinine, angiotonin receptor blocker/angiotension converting enzyme inhibitor, *β*-blocker, inotropes or vasopressors, continuous renal replacement therapy and ventilation (**A**) and cubic spline models in male and female (**B**).

In the fully adjusted restricted cubic spline model, a nonlinear association between sACR and the risk of all-cause mortality was observed (*p* for nonlinear < 0.001) (Figure 3A), and this relationship was consistent across different gender categories (Figure 3B). Additionally, by utilizing a two-segment COX regression model and a recursive algorithm, the inflection point of the association between sACR levels and the risk of all-cause mortality was identified as 3.75. Below this threshold, each incremental unit increase in sACR level was linked to an 19.4% reduction in the risk of all-cause mortality (HR: 0.806, 95% CI: 0.743–0.874). Conversely, when sACR level surpassed 3.75, there was no significant alteration in the risk of all-cause mortality (HR: 1.055, 95% CI: 0.977–1.140). Likelihood ratio tests showed that the two-segment COX regression model provided a superior fit for examining the relationship between sACR and the risk of all-cause mortality compared to the single-line COX regression model (likelihood ratio test *P* < 0.001) (Supplementary Table 2).

### 3.5 Mediation analysis

**Figure 4:**
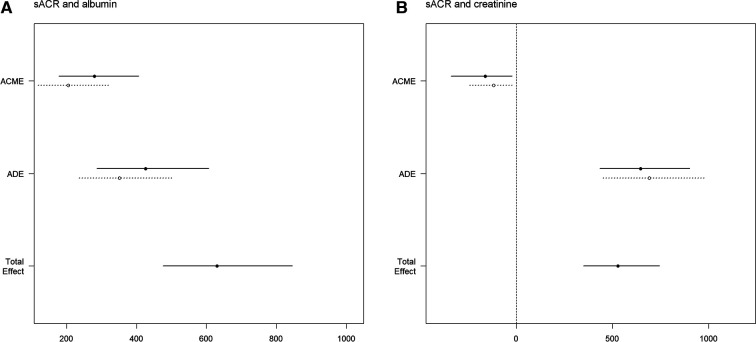
Mediation analysis. Causal mediation analysis for serum albumin (**A**) and serum creatinine (**B**). The solid line represented the mortality group, and the dashed line represented non-mortality group.The ACME and ADE estimates are shown with their confidence intervals. The total effect line demonstrates the overall impact of sACR on mortality, indicating both mediated and direct pathways.

Mediation analyses were conducted between sACR and serum albumin, as well as between sACR and serum creatinine. In the fully adjusted analysis between sACR and serum albumin, the total effect, direct effect, and indirect effect were calculated as 630.92 (95% CI, 475.59∼846.17, *p* < 0.001), 388.48 (95% CI, 263.13∼556.70, *p* < 0.001), and 242.44 (95% CI, 148.08∼361.48, *p* < 0.001), respectively. The indirect effect accounted for 37.9% (95% CI, 0.265–0.530, *p* < 0.001) of the total effect (Figure 4A). In the analysis between sACR and serum creatinine, the total effect, direct effect, and indirect effect were determined to be 529.49 (95% CI, 350.26∼744.66, *p* < 0.001), 669.08 (95% CI, 442.54∼940.53, *p* < 0.001), and −139.59 (95% CI, −287.74∼−19.08, *p* = 0.016), respectively. The indirect effect accounted for −25.7% (95% CI, −0.624∼−0.040, *p* = 0.016) of the total effect (Figure 4B).

### 3.6 Sensitivity analysis

Modified Poisson regression was used as sensitivity analysis. Regression results demonstrated that sACR quartiles remained negatively associated with the risk of all-cause mortality [risk ratio (RR): 0.90, 95% CI 0.86∼0.94] (Supplementary Table 3).

## 4 Discussion

This study highlighted a a significant inverse association between sACR and the risk of all-cause mortality in ICU heart failure patients within one year, demonstrating a threshold effect. Subsequent analysis indicated that sACR, independent of serum albumin and creatinine, directly affected survival outcomes.

Prior studies have shown that sACR is associated with adverse outcomes in cardiovascular diseases. Huang et al. observed that decreased sACR levels were associated with an elevated risk of major adverse cardiac events in patients with ST-segment elevation myocardial infarction (STEMI) undergoing percutaneous coronary intervention (8). This discovery aligns with prior research conducted by Erdems et al., which similarly identified a negative association between sACR levels and adverse outcomes in STEMI patients (5). Shen et al. determined that sACR was a reliable prognostic indicator for heart transplant recipients (9). Additionally, Nseir et al. revealed that sACR levels could predict short-term all-cause mortality in patients with difficult-to-treat Clostridium difficile-associated diarrhea (10). Inspired by these studies, this study investigated the association between sACR and the risk of all-cause mortality in ICU heart failure patients. Utilizing Kaplan-Meier survival analysis, multivariate Cox regression models with varying degrees of adjustment, and modified Poisson regression (sensitivity analysis) with varying degrees of adjustment, our findings consistently indicated that sACR served as a significant stratification factor in this population (Figure 2A, Table 2, Supplementary Table S3). These results suggest that sACR could serve as a valuable prognostic indicator in ICU heart failure patients and may have implications for clinical decisions and long-term management.

Recent research findings have been similar to ours, indicating that low sACR was an independent risk factor for mortality in heart failure patients (7). Nevertheless, different from this study, our study focused on heart failure patients with more severe conditions. Our study revealed a negative, but non-linear relationship between sACR and the risk of all-cause mortality in ICU heart failure patients, characterized by a saturation effect. Notably, sACR levels below the critical threshold of 3.75 exhibited a stronger association with patient outcomes, while variations in sACR levels above this threshold do not significantly impact prognosis. Therefore, to a certain extent, our findings have very important expansions for the above research. These findings provided a more precise understanding of the dose-response relationship between sACR and the risk of all-cause mortality, potentially aiding clinicians in risk stratification.

ICU patients with heart failure often experience a range of comorbidities, including acute kidney injury (AKI) and chronic renal failure, which could significantly affect clinical outcomes. Recent studies have highlighted the need for typing in heterogeneous populations to improve prognostic accuracy (11). Therefore, in this study, we conducted subgroup analyses to account for some of the heterogeneity, examining the impact of sACR across various demographic and clinical characteristics. The prognostic impact of sACR on all-cause mortality was consistent across subgroups, further demonstrating the robustness of this indicator (Figure 2B). However, in the future, further stratification including detailed subtype typing based on AKI status, may provide deeper insights into the prognostic utility of sACR.

Mediation analysis is a statistical technique employed to investigate the underlying mechanisms by which independent variables influence dependent variables. This method aids in discerning whether the impact of the independent variable on the dependent variable is direct or mediated by intermediary variables (albumin and creatinine in this study). The findings of the mediation analysis indicate that sACR influences mortality not only through the pathways involving serum albumin and creatinine, but also through direct effects. The specific mechanism underlying the association between sACR and cardiovascular disease remains unclear. It is clear, however, that sACR is an index composed of two important prognostic factors for cardiovascular disease: serum albumin, which is related to liver function, and serum creatinine, which is related to kidney function. More importantly, the integration of serum albumin and creatinine into sACR has been shown to have a significant impact on the risk of all-cause mortality in heart failure, partially through its direct impact. In addition, findings from the multivariable Cox regression model further suggested that the impact of sACR on outcomes was independent of serum albumin and creatinine. Therefore, integrating albumin and creatinine into sACR may more comprehensively reflect the pathophysiological changes of heart failure and have enhanced prognostic value. Based on previous clinical and preclinical research evidence, we speculate that the direct effect of sACR levels on the risk of all-cause mortality in heart failure patients may be through the these pathways. Firstly, low sACR may indicate malnutrition, and heart failure and malnutrition could be mutually causal, forming a vicious cycle (12). Secondly, low sACR may indicate an inflammatory response, and inflammatory could hinder albumin synthesis, accelerate albumin metabolism, and lead to pathological changes including cardiomyocyte apoptosis and ventricular remodeling (13). Thirdly, low sACR may suggest neuroendocrine activation, particularly the persistent activation of the renin-angiotensin-aldosterone system and the sympathetic nervous system, which could lead to increased protein catabolism, hypoalbuminemia and elevated creatinine levels (14). Further investigation is needed to fully understand the pathological significance of sACR, with potential implications for optimizing secondary prevention strategies.

However, this study was not without limitations. First, this study was a *post hoc* analysis of a retrospective cohort study. Although we used multivariate models to control for confounding variables, certain important risk factors were not included due to data limitations, such as information on prior cardiovascular disease, serum natriuretic peptides, left ventricular ejection fraction at admission, and treatment received during follow-up after discharge. In particular, chronic kidney disease, as a crucial covariate, was not included in this analysis. These may pose a few challenges to the exploration of this study to some extent. Second, it is difficult to tell whether or not the heart failure is the primary cause of ICU admission in MIMIC-Ⅳ. However, ICU admissions are often driven by critical conditions of multiple organs, including heart failure. Third, serum albumin and creatinine levels were measured within the first 24 h of ICU admission. These initial values may not capture changes in renal function or albumin levels that could occur later during the ICU stay. Finally, due to database limitations, we did not continuously monitor changes in covariates and therefore could not perform time-varying covariate analysis. As highlighted in a recent study (15), analysis of time-varying covariates could provide more comprehensive information. Going forward, studies need to be prospectively designed, data collection strategies optimized, and physiological parameters dynamically recorded in cohort populations to obtain more comprehensive assessments and higher clinical benefits.

## 5 Conclusion

The study found that low levels of sACR were independently associated with an increased risk of one-year all-cause mortality in ICU heart failure patients, with a threshold effect, which could potentially serve as an early warning indicator for high-risk populations.

## Data Availability

The datasets presented in this study can be found in online repositories. The names of the repository/repositories and accession number(s) can be found below: https://physionet.org/content/mimiciv/2.2/.
