# Supplementary material for: Association between serum albumin creatinine ratio and all-cause mortality in intensive care unit patients with heart failure
Source: Front Cardiovasc Med. 2024 Jul 4;11:1406294. doi: 10.3389/fcvm.2024.1406294 (PMC11254761; doi:10.3389/fcvm.2024.1406294)
Supplement: Supplementary file 1 [file Table1.docx]

**Supplementary Table 1**. ICD-9 and ICD-10 codes used to screen of patients with congestive heart failure.

| ICD-9 | | | ICD-10 |
| --- | --- | --- | --- |
| 428 | 4258 | 40411 | I43 |
| 4280 | 4259 | 40413 | I50 |
| 4281 | 4284 | 40491 | I099 |
| 4282 | 42840 | 40493 | I110 |
| 42821 | 42841 |  | I130 |
| 42822 | 42842 |  | I132 |
| 42823 | 42843 |  | I255 |
| 4283 | 4289 |  | I420 |
| 42830 | 39891 |  | I425 |
| 42831 | 40201 |  | I426 |
| 42832 | 40211 |  | I427 |
| 4254 | 40291 |  | I428 |
| 4255 | 40401 |  | I429 |
| 4257 | 40403 |  | P290 |

**Supplementary Table 2.** Threshold effect analysis of the relationship between serum albumin creatinine ratio and all-cause mortality.

|  | Threshold of sACR | HR 95CI% | p value |
| --- | --- | --- | --- |
| 1-line cox regression model | - | 0.917(0.873~0.964) | 0.001 |
| 2-line cox regression model | < 3.75 | 0.806(0.743~0.874) | < 0.001 |
|  | ≥ 3.75 | 1.055(0.977~1.140) | 0.173 |
| Likelihood Ratio test | - | | < 0.001 |

Data were adjusted for age, gender, diabetes, hypertension, chronic obstructive pulmonary disease, neutrophils, hemoglobin, albumin, total bilirubin, blood urea nitrogen, creatinine, angiotensin-converting enzyme inhibitor / angiotensin Ⅱ receptor blockers, β-blockers, inotropes or vasopressors, continuous renal replacement therapy and ventilation.

**Supplementary Table 3.** Modified Poisson regression models for the association between serum albumin creatinine ratio and all-cause mortality in one year.

| sACR | Case/Total | Model 0 | Model 1 | Model 2 | Model 3 |
| --- | --- | --- | --- | --- | --- |
| Quartiles |  | Risk Ratio | | | |
| Q1 | 1126/4506 | ref | ref | ref | ref |
| Q2 | 1127/4506 | 0.83(0.77,0.90) | 0.77(0.71,0.83) | 0.87(0.79,0.95) | 0.89 (0.81,0.98) |
| Q3 | 1126/4506 | 0.72(0.65,0.78) | 0.67(0.61,0.73) | 0.82(0.73,0.92) | 0.85(0.75,0.95) |
| Q4 | 1127/4506 | 0.53(0.48,0.59) | 0.52(0.47,0.57) | 0.68(0.59,0.79) | 0.72(0.62,0.83) |
| p for trend |  | < 0.001 | < 0.001 | < 0.001 | < 0.001 |
| Per quartiles increase |  | 0.82(0.79,0.84) | 0.81(0.78,0.84) | 0.89(0.85,0.93) | 0.90 (0.86,0.94) |
| Per unit increase |  | 0.87(0.84,0.89) | 0.86(0.83,0.88) | 0.94(0.90,0.97) | 0.94(0.91,0.98) |

Model 0: serum albumin creatinine ratio without adjust; Model 1: age, gender, diabetes, hypertension and chronic obstructive pulmonary disease were adjusted; Model 2: the variables in model 1 plus neutrophils, hemoglobin, albumin, total bilirubin, blood urea nitrogen, creatinine were adjusted; Model 3: the variables in model 2 plus angiotensin-converting enzyme inhibitor / angiotensin Ⅱ receptor blockers, β-blockers, inotropes or vasopressors, continuous renal replacement therapy and ventilation were adjusted.
